# Study of the mechanical properties and propagation mechanisms of non-coplanar and discontinuous joints via numerical simulation experiments

**DOI:** 10.1038/s41598-024-63576-w

**Published:** 2024-06-14

**Authors:** Yuanming Liu, Lankai Ya, Wei Wang, Qingzhi Chen, Zhongxing Wang, Zhaolei Teng, Jiajun Cheng

**Affiliations:** 1 College of Civil Engineering, Guizhou University, Guiyang, 550025 China; 2 Guizhou Provincial Key Laboratory of Rock and Soil Mechanics and Engineering Safety, Guiyang, 550025 China; 3 China Construction Third Engineering Bureau Group Co., Ltd., Wuhan, China

**Keywords:** Discontinuous joint, Mechanical properties, Propagation mechanisms, Direct shear test, Environmental sciences, Engineering, Mathematics and computing

## Abstract

Non-coplanar and discontinuously jointed rock masses are more complex than coplanar and discontinuously jointed rock masses. The mechanical properties and propagation mechanisms of non-coplanar and discontinuous joints were studied via direct shear tests with microscopic numerical simulation experiments. The numerical simulation tests were performed under different normal stresses, joint inclination angles, and shear rates. The numerical experimental results show that the microscale failure of non-coplanar and discontinuously jointed rock masses is mainly caused by tensile cracks. Additionally, when the peak shear stress is reached, the growth rate of cracks increases rapidly, and the number of cracks increases with increasing normal stress. The shear strength of non-coplanar and discontinuously jointed rock masses increases with increasing normal stress. Under the same normal stress, the variation curves of the shear strength of non-coplanar and discontinuously jointed rock masses with respect to the dip angle exhibit an “S”-shaped nonlinear pattern. Rock masses with joint inclination angles of approximately 15° and 65° have minimum and maximum shear strengths, respectively. The joint dip angle has a significant impact on the final failure mode of rock bridges in the rock mass. As the joint dip angle increases, the final failure modes of rock bridges change from “end-to-end” connection to a combination of “head-to-head” and “tail-to-tail” connections. The shear rate has a certain impact on the peak shear stress, but the impact is not significant. The spatial distribution of the tensile force chains changes as shearing progresses, and stress concentration occurs at the tips of the original joints, which is the reason for the development of long tensile cracks in the deeper parts.

## Introduction

Non-coplanar and discontinuous nodular rock masses are widely encountered in mining tunnels and rocky slopes, and the existence of joints makes such rock masses discontinuous and inhomogeneous. Under the condition of direct shear, different normal stresses and joint arrangements affect the failure mode and mechanical properties of a rock mass^1^. The failure modes of rock masses usually include joint and rock bridge damage, and the presence of rock bridges leads to qualitative changes in both the force characteristics and the damage mode of the rock masses^2^. Therefore, it is important to study the damage mechanism of discontinuously jointed rock masses and the damage mode of rock bridges to elucidate instability in rock engineering.

Since rocks are often subjected to compressive shear, the damage mode of discontinuously jointed rock masses can be studied via direct shear tests. Lajtai et al.^3^ conducted direct shear tests on coplanar discontinuous joints with different normal stresses and found that cracks were first generated at the ends of the joints, that the expansion direction of the cracks did not coincide with the shear direction, and that the final damage mode was shear damage. By performing direct shear tests on rock-like materials, Wang et al.^4^, Bai et al.^5^, and Shen et al.^6^ found that the joint connectivity rate, roughness, arrangement mode, normal stress and loading rate of the joint had important impacts on the penetration failure mode of the discontinuously jointed rock-like materials through direct shear tests. Liu et al.^7^ conducted a large number of direct shear tests on coplanar, closed, and discontinuously jointed nodular rock masses to verify that the tangential deformation curve has segmental characteristics and can be divided into four stages: the prefracture stage, stable extension stage, unstable extension stage, and residual stage. Tang et al.^8,9^ investigated the peak shear strength of rock joints under various contact conditions and proposed a new shear strength criterion. They also explored the effects of cyclic freeze‒thaw cycles and wet‒dry cycles on the shear strength of jointed rock masses and found that both environmental factors have a diminishing effect on the peak shear stress of the rock mass.

At present, there are more studies on jointed rock masses in terms of physical experiments or numerical simulation experiments, and there are fewer studies on the final failure mechanism of non-coplanar discontinuously jointed rock masses^1,10,11^. The reason for this difference is that the preparation of non-coplanar discontinuously jointed specimens is more difficult than that of coplanar discontinuously jointed nodular rock masses, physical experiments can only provide an indication of the damage pattern, and it is difficult to analyze the cause of the damage pattern, especially the development of cracks, at small scales.

With the continuous development of computer technology, numerical simulation has become an important auxiliary tool in the field of geotechnical engineering. Numerical simulation can fully reproduce the stresses in a specimen and is economical, efficient and reproducible. In terms of coplanar jointed rock masses, Liu et al.^12^ designed direct shear tests of coplanar discontinuous joint models with different connectivity rates and normal stresses, performed numerical simulations using granular flow software, and found that the shear strength of coplanar discontinuously jointed rock masses was mainly provided by rock bridges. Chen et al.^13^ studied the effects of different joint undulation angles on the strength and deformation characteristics of jointed rock masses under the same normal stress conditions and found that the joint undulation angle had an important effect on the shear strength and tensile fracture development of rock masses. Yu et al.^14^ introduced strength weakening factors in CPM media for simulating weathering leading to rock strength weakening effects, and with the help of particle flow soft PFC^2D^, they found that the weakening effect could lead to rapid crack development within the specimen. Zhou et al.^15–20^ analyzed the mechanical change patterns and damage mechanisms during the direct shear of jointed rock masses from scales up to the macroscale. In terms of discontinuously jointed rock masses, Zhao et al.^21^ conducted direct shear tests on fabricated model materials and found that the shear stress curves also exhibited obvious segmental characteristics. Jiang et al.^22^ performed DEM numerical simulations of non-coplanar and discontinuous nodular rock masses and explained the changes in the mechanical properties of the rock masses through the distribution of interparticle pressure chains. Ghazvinian et al.^23^ studied the propagation of tensile cracks in rock-like materials and found that an increase in joint length decreases the tensile strength, while a reduction in ligament length increases the tensile strength. Moreover, the Brazilian test was found to overestimate the tensile strength. Haeri et al.^24^ used PFC^2D^ simulations to reveal the significant impact of rock joint opening on the formation of shear bands and the failure behavior of rock bridges. Through both physical and PFC^2D^ simulation experiments, Sarfarazi et al.^25,26^ conducted research on the impact of joint number, angle, and separation on the shear failure of rock bridges. It was found that joint characteristics significantly influence the failure mode and strength of rock bridges and that the formation of shear bands is closely related to the arrangement of joints. Fu et al.^27^ explored the impact of joint overlap on the shear behavior of rock bridges through experiments and numerical simulations and discovered that joint overlap reduces shear strength and leads to a transition in failure mode from progressive failure to brittle failure with increasing normal stress. Chen et al.^28^ carried out numerical simulations of nonpenetrating joints under different confining pressure conditions and reported that the development of tensile fractures decreased with increasing confining pressure. Discrete elements can easily handle discontinuous medium mechanics and effectively reflect discontinuous processes such as cracking and separation of the simulated medium^29^.

In this paper, the discrete element software PFC^2D^ is used to conduct numerical simulations of non-coplanar and discontinuous nodular rock masses under different normal stresses, different nodal dips, and different shear rates to study their small-scale expansion mechanisms.

## Model development and experimental protocol design

### Determination of the interparticle contact model

When using PFC modeling, the particles are rigidly connected to each other by default. The different contact modes and mechanical properties between the particles are the most critical part of the modeling and determines the basic mechanical properties of the whole model. The linear contact bond model provides a very small linear elastic mechanical contact that does not resist the relative rotation among the contact surfaces of the particles and cannot carry frictional forces. The contact interface of the linear parallel bond model is a bonding interface with a section size that can transfer either force or moment. The interparticle forces are reflected through the contact force chain. When the local stress exceeds the linear parallel bonding strength, the contact will undergo bond rupture, forming microcracks. Therefore, the linear parallel bond model can effectively simulate the mechanical properties of rock materials^30,31^.

For the generation of joints, the SRM (synthetic rock mass) method in PFC 5.0 software can be used^32^; the generated “joint” is just a line in the rock mass and does not have the physical properties of a real joint. In this case, the program will automatically recognize this line and replace the contact model of the surrounding particles with a smooth joint model and give it mechanical parameters so that it has the shape and properties of a real joint^33,34^.

**Figure 1:**
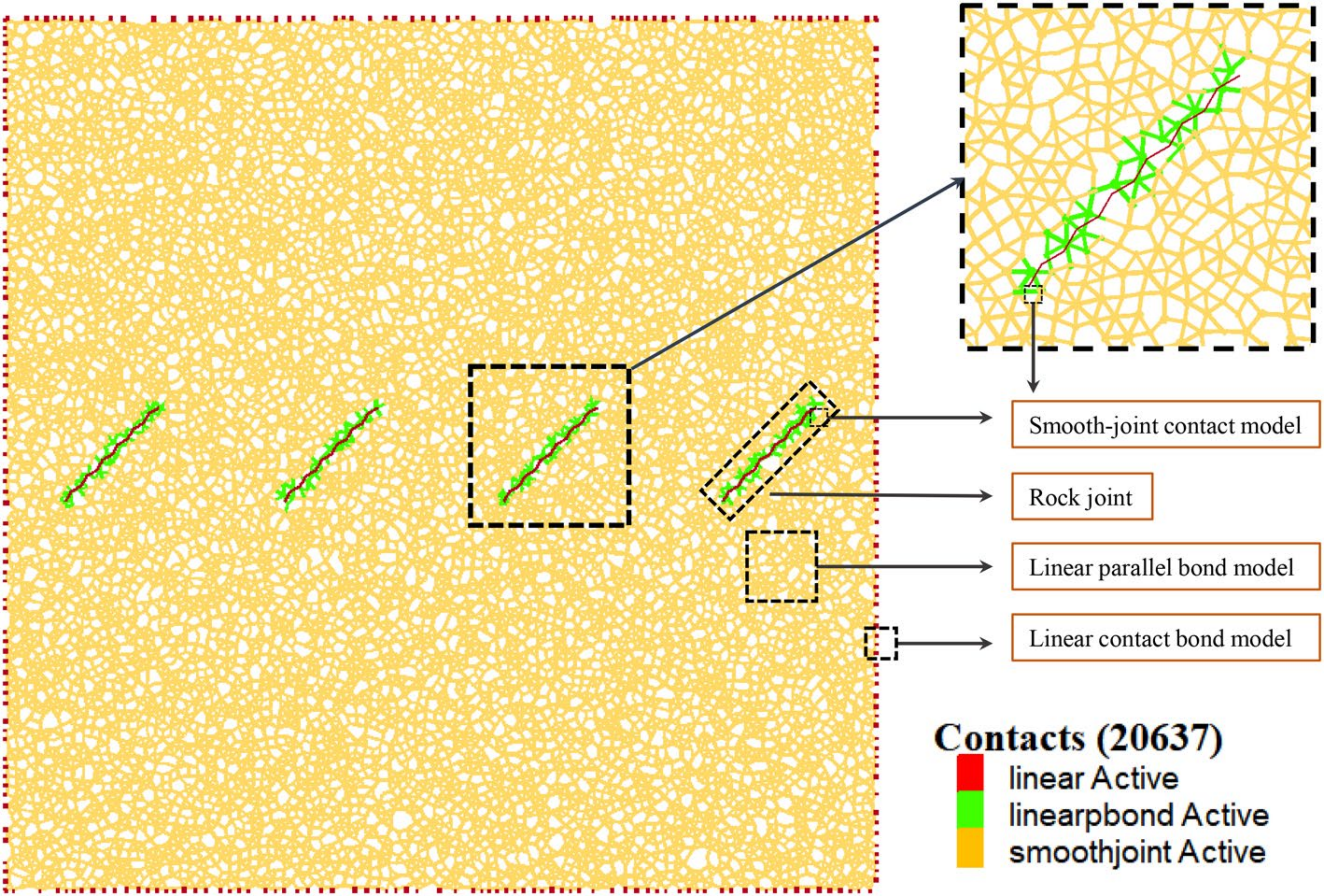
The numerical model.

Consequently, this study utilizes the linear contact bond model to simulate the interactions between particles and the wall, denoted by red lines; the linear parallel bond model to represent the contacts between particles, denoted by orange lines; and the smooth joint model to emulate the joints within the rock mass. The lines generated by the smooth joint model at the interface with the surrounding particles are marked with green lines. The details are shown in Fig. 1.

### Determination of mesoscale parameters

This paper employs cement mortar with a mixing ratio of cement:sand:water = 2:3:1 to simulate rock material. The physical and mechanical parameters of the rock-like material are as follows: density, 2.65 kg/m^3^; compressive strength, 46.86 MPa; tensile strength, 3.51 MPa; elastic modulus, 8.75 GPa; Poisson’s ratio, 0.25; cohesive force, 5.25 MPa; and friction angle, 46°. To obtain mechanical properties similar to those of real jointed rock masses, it is necessary to change the mesoscale parameters (particle stiffness ratio, contact modulus, bond strength, friction coefficient, etc.) and use PFC2D software to carry out a series of experiments similar to laboratory tests: uniaxial compression tests, Brazilian splitting tests, direct tensile tests and other tests.

**Figure 2:**
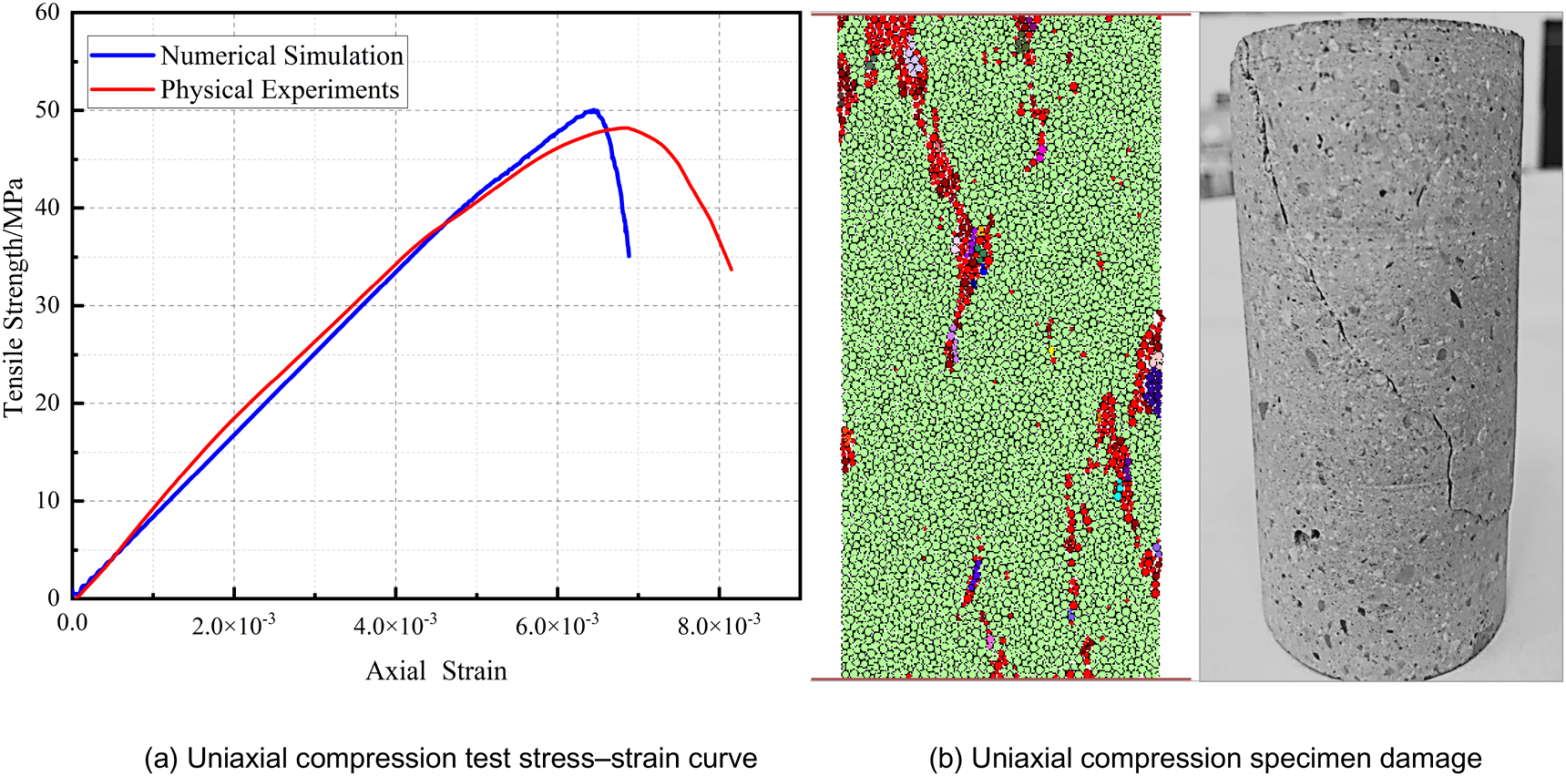
Uniaxial compression test stress–strain curves and failure diagrams of the specimens from the physical experiment and numerical simulation.

Uniaxial compression tests are used to calibrate the uniaxial compressive strength and elastic modulus of a model. Figure 2 presents the uniaxial compressive stress‒strain curves and failure diagrams from the physical experiment and numerical simulation. Figure 2 shows that the uniaxial compressive strengths of the specimens obtained from the physical experiment and numerical simulation test are 50.09 MPa and 46.86 MPa, respectively, which is a difference of 6.45%. The elastic moduli are 8.13 GPa and 8.75 GPa, respectively, with a discrepancy of 7.09%.

**Figure 3:**
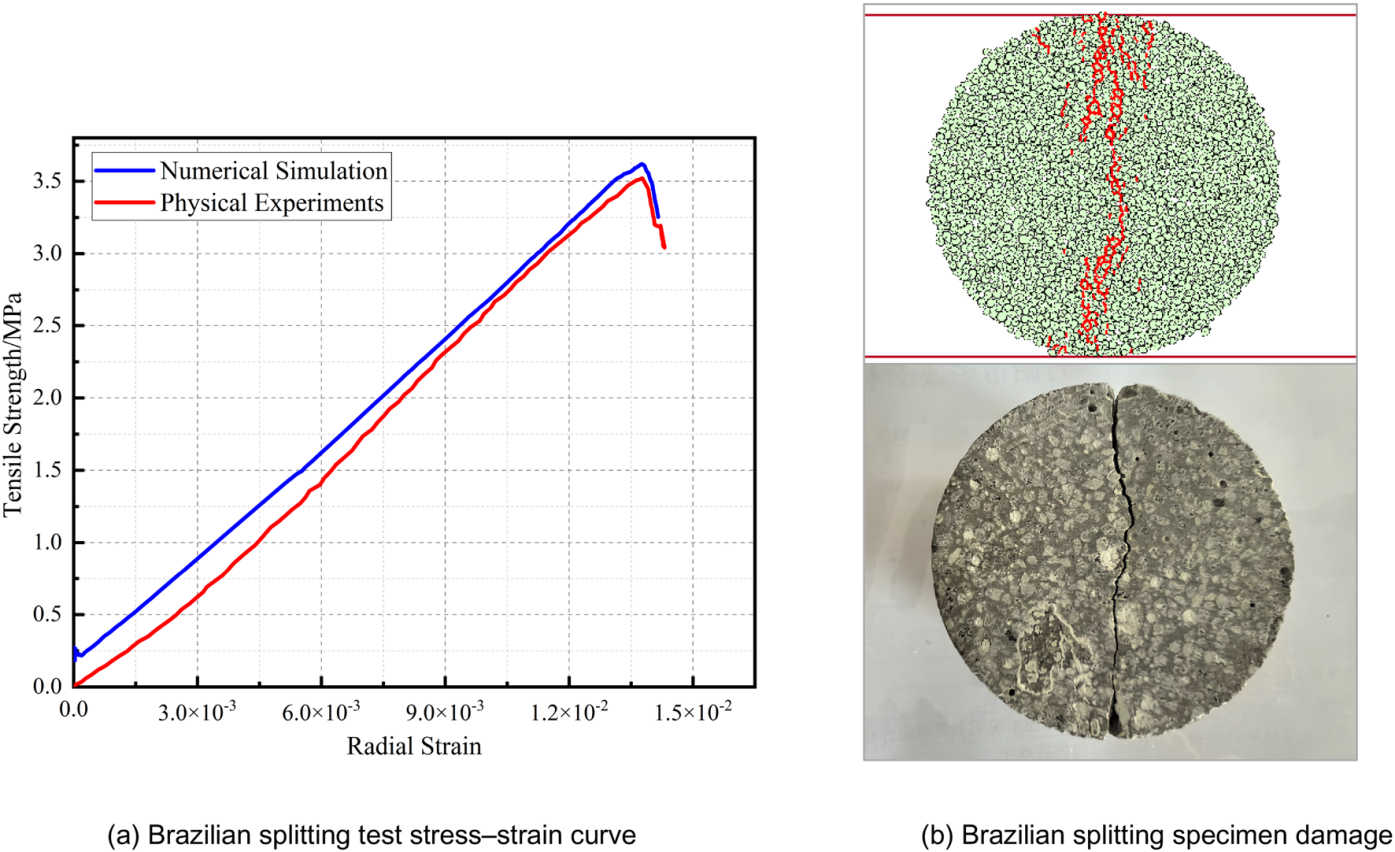
Stress‒strain curves and failure diagrams of the Brazilian test specimens from the physical experiment and numerical simulation.

The Brazilian splitting test is used to check the tensile strength of the specimen. Figure 3 shows the Brazilian tensile stress‒strain curves and failure diagrams of the specimens. Figure 3 shows that the tensile strengths of the specimens in the two types of tests are 3.62 MPa and 3.51 MPa, respectively, with a difference of 3.04%.

The parameters obtained from physical experiments and numerical simulations are shown in Table 1. Therefore, the mechanical property results obtained by using PFC2D to establish model specimens are in good agreement with the results of laboratory tests conducted by Liu et al.^35,36^, which ensures that the established numerical model has application value for the study of the mechanical properties of discontinuously jointed rock masses.

**Table 1:** The results of the physical tests and numerical simulations.

Mechanical parameters	Physical experiments	Numerical simulations	Error/%
*E*/GPa	8.75	8.13	7.1
μ	0.25	0.24	4
*σ*_c_/MPa	46.86	50.09	6.45
*σ*_t_/MPa	3.51	3.62	3.04

The final mesoscale mechanical parameters of the PFC simulations are shown in Table 2.

**Table 2:** PFC^2D^ micromechanical parameters of model specimens.

Parameters of the particles								
Particle density								
/kg·m^-3^	Model porosity	Particle contact modulus/GPa	Friction coefficient	Particle stiffness ratio	Normal bonding strength/MPa	Tangential bonding strength/MPa	Parallel bonding stiffness ratio	Parallel modulus of cohesion/GPa
2650	0.02	4.3	0.57	3.2	12	34	3.2	4.3

Partial parameters of the wall	Parameters of the smooth joints							
Normal stiffness/N m^−1^	Normal stiffness/N m^−1^	Normal stiffness/N m^−1^	Normal stiffness/N m^−1^	Friction coefficient	Joint thickness/mm			
1.0 × 10^10^	1.0 × 10^8^	2.0 × 10^9^	2.0 × 10^9^	0.2	1.0			

### Numerical simulation test program

**Figure 4:**
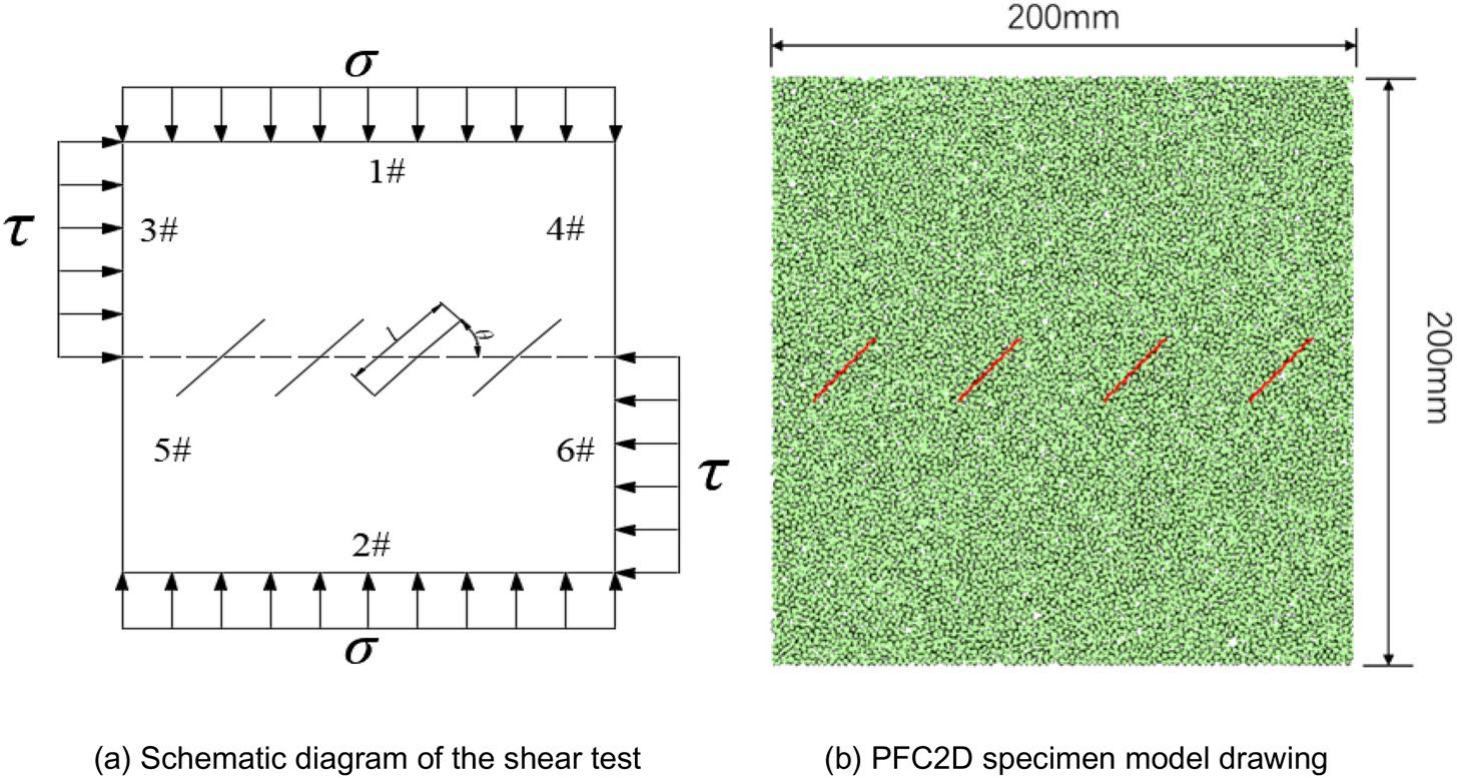
Non-coplanar discontinuous joint layout.

First, 6 walls are defined by the starting point coordinates, and the range surrounded by the walls is 200 mm × 200 mm. By setting the porosity to 0.02, the particles in the model specimen are uniformly distributed, and the radii of the particles are Rmin = 0.87 mm and Rmax = 1.44 mm in the given range, which are cogenerated into 9143 particles. The #1, #3, and #4 walls form the upper half of the shear box, and the #2, #5, and #6 walls form the lower half of the shear box. The #5 and #6 walls form the lower half of the shear box, and the lower half of the shear box is made stationary during the servo process so that the upper half moves to the right at a constant shear rate. For the internal arrangement of the four non-coplanar joints along a shear surface, the center points of the joints on the shear surface are shown in Fig. 4.

Figure 4 is a schematic diagram of the PFC specimen, where the length of a joint *l* is 30 mm, and the joint dip angle *θ* is 45°. To study the effects of different normal stresses *σ,* different joint dips* θ* and different shear rates* v* on the strength and joint growth pattern of the discontinuously jointed rock mass, the specimen simulation scheme is shown in Table 3.

**Table 3:** Numerical simulation scheme for a non-coplanar discontinuously jointed rock mass.

Test conditions	Normal stress σ/MPa	Inclination of the joints θ/°	Length of the joints *l*/mm	Shear rate/mm s^−1^	Running timestep/step	Note
Different normal stresses	1.0–5.0	45	30	0.06	2.3 * 10^5^	5 sets of normal stress
Different nodal inclinations	1.0–5.0	0–90	30	0.06	2.3 * 10^5^	81 sets of nodal inclinations
Different shear rates	1.0	60	30	0.02–0.1	1.8 * 10^5^–5.7 * 10^5^	5 sets of shear rates

12–2 Particle force chain and crack development diagrams at different shear displaceme.

## Results and analysis

### Effect of the normal stress

**Figure 5:**
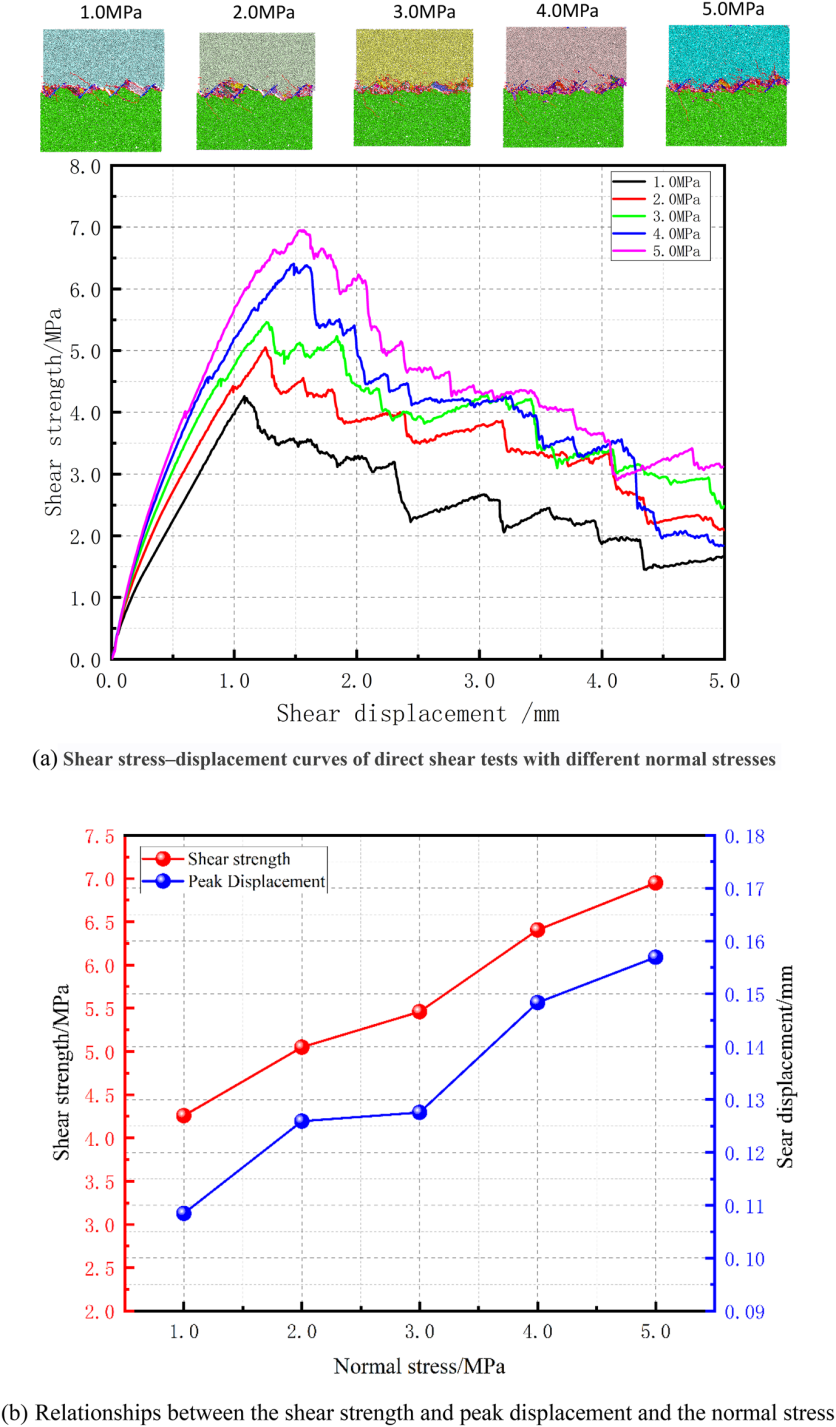
Mechanical behavior during direct shear tests under different normal stresses.

Figure 5 shows the shear stress‒shear displacement curves and the specimen failure diagrams obtained under the conditions of five levels of normal stress, a joint dip angle of 45°, a joint length of 30 mm, and a shear rate of 0.06 mm/s in a direct shear test.

When the normal stress is 1.0 MPa, 2.0 MPa, 3.0 MPa, 4.0 MPa, and 5.0 MPa, the corresponding peak shear stresses are 4.258 MPa, 5.049 MPa, 5.46 MPa, 6.406 MPa, and 6.95 MPa, respectively; the corresponding peak displacements (defined as the shear displacement corresponding to the peak shear stress) are 1.08 mm, 1.26 mm, 1.27 mm, 1.48 mm, and 1.57 mm, respectively. The slope of the peak shear stress curve is consistent with that of the peak displacement curve, and both show a nonlinear increasing trend with increasing normal stress (see Fig. 5b). By comparing the specimen failure diagrams under the conditions of five levels of normal stress, it is found that the joint development of the rock mass is different under different normal stresses. When the normal stress is less than 3 MPa, the joints exhibit a significant climbing phenomenon under direct shear, which is specifically manifested by the appearance of large cracks in the area along the line connecting the loading end and the fixed end in the shear direction. In contrast, when the normal stress is greater than or equal to 3 MPa, the climbing phenomenon is not pronounced, and the failure surface is more fragmented. This indicates that under the condition of a 45° joint dip angle, an increase in the normal stress suppresses the climbing effect triggered by the direct shear action on the specimen.

**Figure 6:**
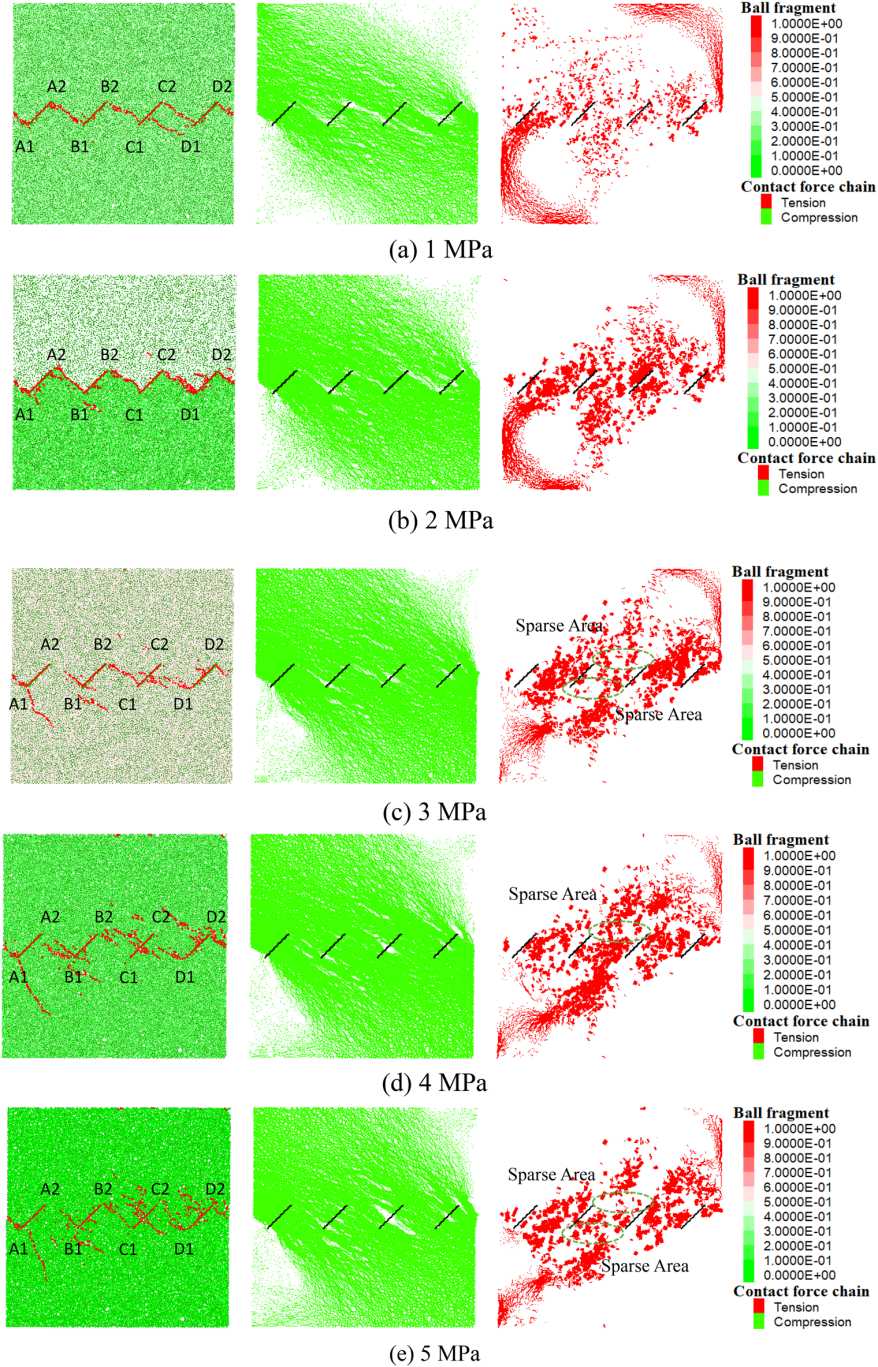
Interparticle force chain diagram of different normal stresses at the peak stress.

Figure 6 presents the peak shear stress corresponding to the crack distribution and particle force chain diagrams under the conditions of five levels of normal stress (from left to right, the first diagram is the crack distribution, the second is the compressive force chain, and the third is the tensile force chain). In the crack distribution diagram, both the original and new joints are uniformly represented by red lines. In the first diagram for each level of normal stress, the heads and tails of four joints are marked, where A1, B1, C1, and D1 represent the heads of the first to fourth joints from the left, respectively, while A2, B2, C2, and D2 represent the tails of the first to fourth joints from the left, respectively. In the force chain diagrams, the green lines indicate compressive force chains, the red lines represent tensile force chains, and the black lines denote the original joints. The naming convention is consistent with that of the crack distribution diagram and will not be repeated here.

Here, A2–B1 is defined as the connection between the tail of joint A and the head of joint B, and B2–CC represents the connection between the tail of joint B and the middle of joint C, with other cases being similar.

The crack distribution diagrams corresponding to the peak shear stress of the specimens under five levels of normal stress are compared. The following observations were made: (1) When the normal stress is 1 MPa and 2 MPa, the rock bridge between joints exhibits a “head-to-tail” connection pattern of cracks A2–B1, B2–C1, and C2–D1, and at this time, no tensile fissures appear at the lower end of the left joint of the rock mass. (2) When the normal stress is between 3 and 5 MPa, the rock bridge between joints A and B still maintains a “head-to-tail” failure pattern; in addition to the clear “head-to-tail” (B2–C1, C2–D1) failure paths appearing within the rock bridges between joints B and C and C and D, there are also accompanying failure paths from the end to the middle of the adjacent joint (B2–CC, CC–D1) within these two rock bridges. (3) Taking B2–C1 and C2–D1 as examples, with increasing normal stress, the former shows a trend of change from the “head-to-tail connection of B2–C1” at 3 MPa to the “tail-to-tail” connection of B2–C2 at 5 MPa. In contrast, the latter exhibits a trend from the “head-to-tail” connection of C2–D1 at 3 MPa to the “head-to-head” connection of C1–D1 at 5 MPa. Concurrently, under the three levels of normal stress, tensile fissures extending deep into the specimen originate from the head of joint A.

Taking the center of the specimen as the origin of the coordinates, it can be observed from the tensile force chain and compressive force chain diagrams corresponding to the peak shear stress under five levels of normal stress that the green compressive stress chains are all distributed in a centrally symmetric manner. Under low normal stresses (1 MPa, 2 MPa), the tensile force chains each appear as two separate chains distributed within the rock bridges between joints A and B and C and D, exhibiting a centrally symmetric distribution pattern. However, when the normal stress is between 3 and 5 MPa, the tensile force chains within the rock bridges between joints A and B and C and D dissipate in all three rock masses, concentrating instead within the rock bridge between joints B and C. Additionally, dense regions of tensile force chains are located at almost the ends of joints B and C. In the green areas of B2-C2 and C1-D1 in the corresponding figure, sparse tensile force chains are observed. In conjunction with the crack distribution diagram, it can be inferred that the sparse areas are due to the development of cracks, which in turn validates the cause of the rock bridge's throughgoing failure indirectly. This indicates that as the normal stress increases, the development of tensile force chains at the microscale decreases, while macroscopically, this results in the inhibition of tensile crack propagation.

Overall, the force chain lines are thicker in the corresponding figure, indicating that there is a large pressure between the particles, so greater shear stress is required to cause shear displacement of the model specimen.

**Figure 7:**
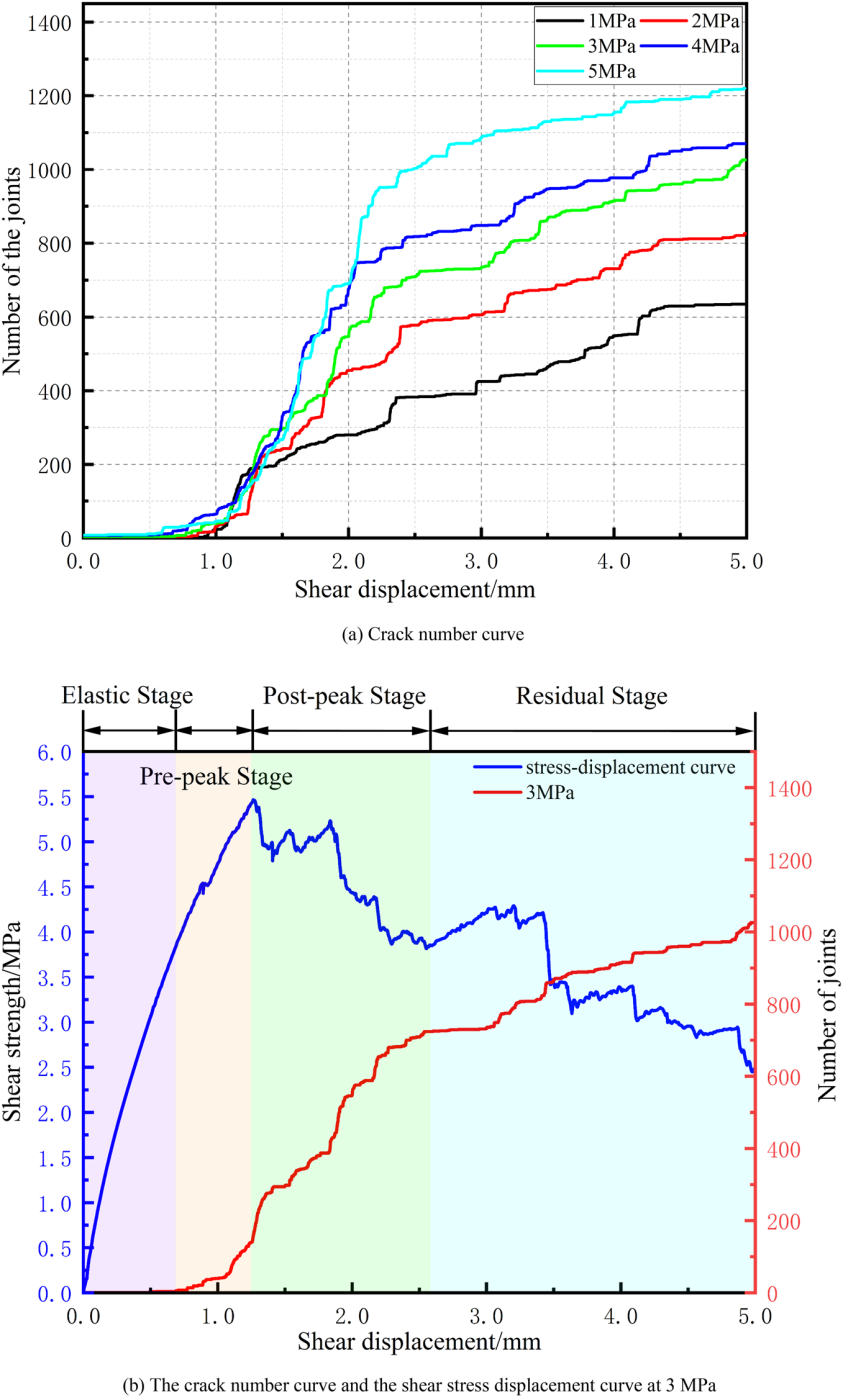
Crack number curves under different stresses and the shear stress‒displacement curve at 3 MPa.

When the rock material is subjected to a certain degree of external load, mesoscale cracks will form inside the rock. The generation of such cracks in the numerical simulation of particle flow is characterized by the parameter setting of the contact model. Shear or tensile cracks occur in parallel bond models when the normal bond strength and tangential bond strength is overcome. To study the distribution and development of cracks under different normal stresses, the occurrence and number of cracks were tracked in numerical simulation experiments. Figure 7 shows the stress‒displacement curve for a normal stress of 3 MPa and the relationship between the number of cracks developed and the shear displacement under the action of five different normal stresses.

Combining the crack number curves from Fig. 7a,b, it can be observed that cracks begin to develop only after a certain amount of shear displacement has occurred in the specimen, and the number of cracks increases nonlinearly with increasing shear displacement. The fastest growth of the crack number curve occurs during the prepeak and postpeak stages. Additionally, both the total number of cracks within the specimen and the steepest slope of the curve increase with increasing normal stress. By comparing the shear stress‒displacement curves in Fig. 5 and the crack number curves in Fig. 7 under the five levels of normal stress tested, it is evident that there is a distinct phased nature to the shear stress‒displacement curves under different normal stress conditions, which can be specifically divided into the linear elastic stage, the prepeak stage, the postpeak stage, and the residual deformation stage.

Due to space limitations, this paper takes the shear stress‒displacement curve and the crack number curve under the condition of 3 MPa of normal stress (as shown in Fig. 7b) as the subject of analysis. Below 0.71 mm, the shear stress is linearly elastic with respect to the shear displacement, indicating that the particles in the model specimen are compacted without the generation of new cracks (the linear elastic stage). As the shear displacement increases from 0.71 to 1.27 mm, the cracks begin to develop slowly, and the number of cracks remains steadily below 150, indicating that the specimen is subjected to the action of low-level shear force, with the cracks developing stably (the prepeak stage). When the shear displacement reaches approximately 1.28 mm, the shear stress reaches its peak, after which there is a sharp decrease in the shear stress‒displacement curve. At this point, the number of developing cracks increases rapidly (the postpeak stage). As the shear displacement continues to increase, the shear curve enters the residual deformation stage. When the shear displacement reaches 0.5 mm, the trend of crack development gradually plateaus, and the number of approaches the maximum.

**Figure 8:**
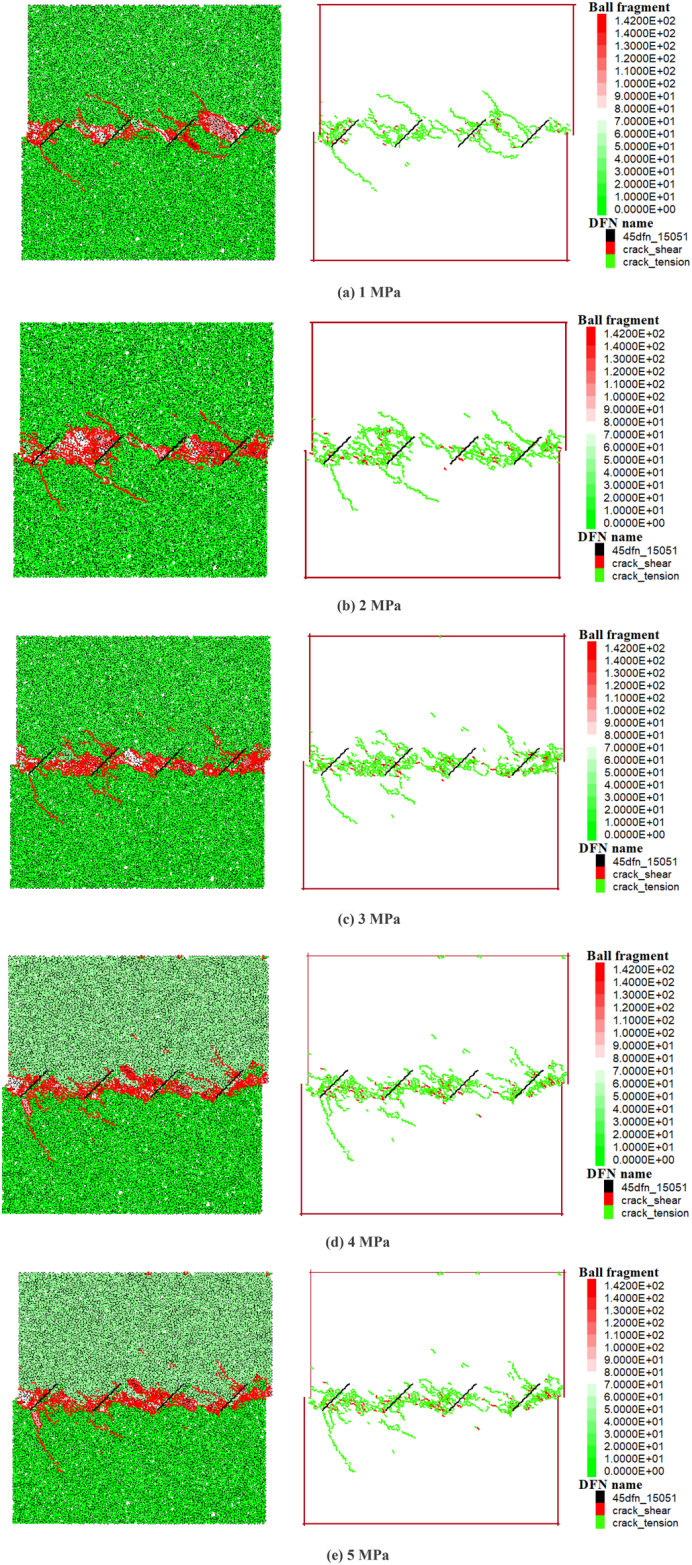
Distribution of cracks under sequential normal stresses.

When the shear displacement reaches 5.0 mm, the distribution of cracks within the model specimen under five different levels of normal stress is shown in Fig. 8. In the specimen block diagram, black lines represent the original joints, red lines represent new joints, and the rest are the particle model. Here, the built-in fragment language function of the PFC is used, where different colors indicate blocks generated at different times. Cracks are mainly distributed on both sides along the shear surface (red is the resulting crack, and the remainder is the particle model; here, the PFC has a fragment language function, and different colors represent blocks generated in different periods). It is evident that under the same shear displacement, the degree of cracking in the shear band area along the line connecting the loading end and the fixed end becomes denser with increasing normal stress. This is also the reason for the increase in the number of blocks along the shear band and the reduction in the volume of large blocks at the microscale. When the normal stress is 5 MPa and the shear displacement reaches 5.0 mm, cracks are more concentrated in the middle of adjacent joints, and a large number of cracks are also generated at the left loading end and the right fixed end.

**Figure 9:**
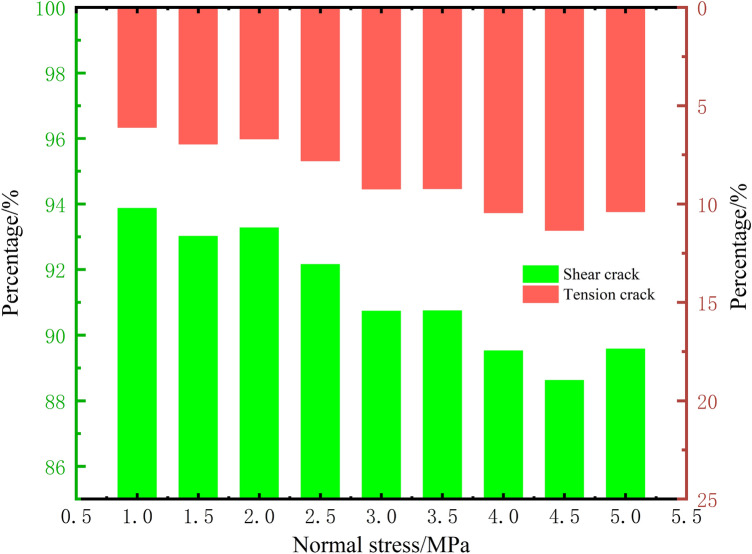
The relationship between the crack development proportion and different normal stresses.

The proportionate relationship between tensile and shear cracks as a percentage of the total number of cracks under different normal stresses is shown in Fig. 9. The number of tensile cracks decreases with increasing normal stress, while the number of shear cracks decreases. The trend of their relationship explains the specimen failure characteristics mentioned earlier; as the normal stress increases, the development of tensile cracks is suppressed, leading to the development of shear cracks along the shear band. The crack development of the specimen concentrates at the shear band along the line connecting the loading end and the fixed end (for details, see the content of Fig. 8 in the previous text).

### Effect of the inclination angle

**Figure 10:**
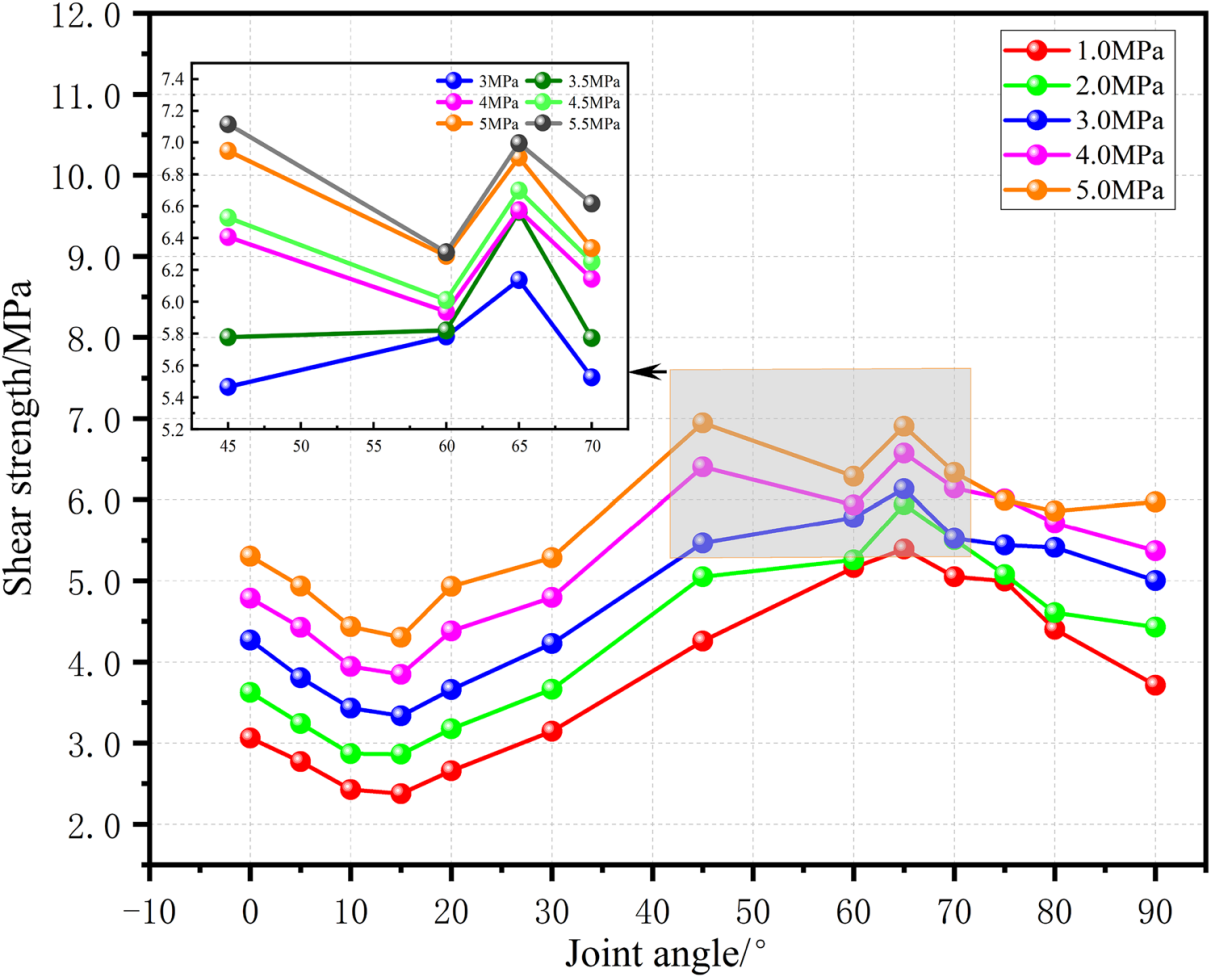
Relationship between the joint inclination angle and peak shear strength under five normal stresses.

Under the conditions of five levels of normal stress (1–5 MPa), the variation in the peak shear stress in the model specimen with respect to the dip angle (0°–90°) is depicted in Fig. 10. All five peak shear stress curves exhibit an “S”-shaped variation with respect to the joint dip angle. The trend of shear strength variation with dip angle (0°–90°) simulated in this paper is similar to the experimental results obtained by Gehle (Reference^37^).

Regarding the minimum shear strength, when the normal stress increases from 1 to 5 MPa, the shear strength of the rock mass is at its minimum at a joint dip angle of 15°, and the joint dip angle corresponding to the minimum shear strength increases with increasing normal stress. When the normal stress is between 1 and 4 MPa, the shear strength of the rock mass is at its maximum at a joint dip angle of 65°. When the normal stress is between 4 and 5 MPa, the curve exhibits two extreme values, and at a normal stress of 5.0 MPa, the peak shear stress occurs in the special case of a joint dip angle of 45°.

Notably, when the normal stress is greater than or equal to 2 MPa, the curve exhibits a concave downward phenomenon within the 45°–65° range. To avoid randomness, the portion of the curve between 3 and 5 MPa (the gray area in the figure) is selected, and normal stresses of 3.5 MPa, 4.5 MPa, and 5.5 MPa are interpolated to study the variation in shear strength of specimens with joint dip angles from 45° to 70° under five levels of normal stress (see the inset in Fig. 10). The results indicate that as the normal stress increases from 3 to 5.5 MPa, the concave downward phenomenon of the curve within the 45°–65° range becomes pronounced. Moreover, when the normal stress exceeds 5 MPa, the shear strength of the specimen with a joint dip angle of 60° is less than that of the specimen with a joint dip angle of 45°.

**Figure 11:**
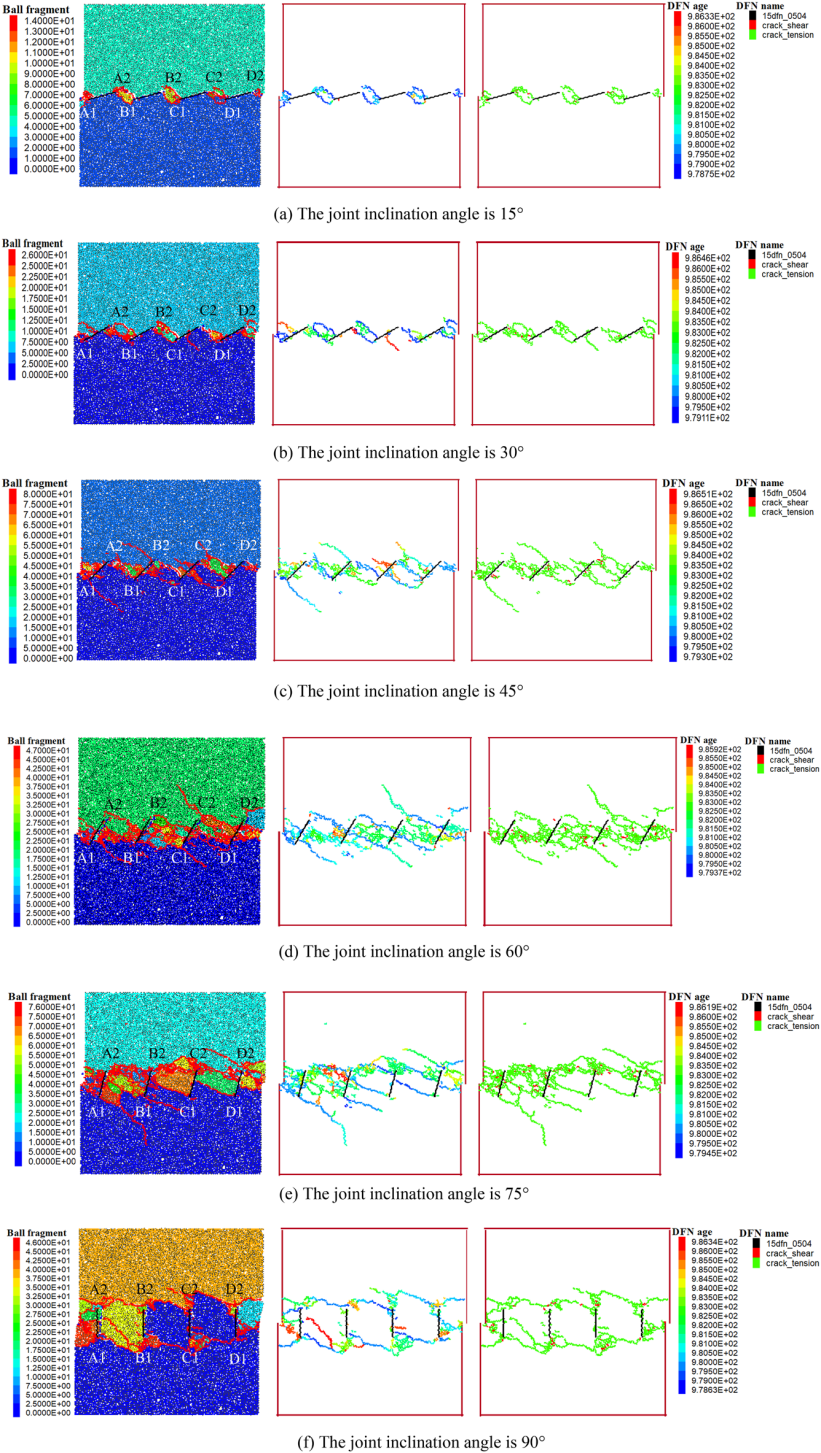
The penetrating failure modes of joints with different inclination angles.

This study investigated the failure modes of rock bridges in specimens with different joint dip angles (15°, 30°, 45°, 60°, 75°, and 90°). The variation in the joint dip angle changes the length of the rock bridges. In Fig. 11, the first diagram of each condition is the final failure diagram of the specimen, with different colors representing blocks generated at different times; the second diagram is the final crack distribution diagram, where different colors represent cracks at various stages; and the third diagram is the tensile crack–shear crack distribution diagram. In Fig. 11, the notation for the failure modes and connection patterns of the original joints are consistent with the previous text.

1. When the joint dip angle is between 15° and 45°, the rock bridges between joints A and B, B and C, and C and D all exhibit a clear failure mode characterized by an “end-to-end” connection (A2–B1, B2–C1, C2–D1) starting from the ends of the original joints and extending toward the adjacent ends of the nearby original joints. According to the third diagram of each condition, the final failure of the rock bridge is predominantly caused by tensile cracks.
2. When the joint dip angle is between 30° and 60°, the crack initiation points shift from the ends of the original joints toward the middle of the model. The final failure mode within the three rock bridges evolves from an “end-to-end” connection pattern (A2–B1, B2–C1 at 30° and 45°) to a connection from joint ends to the middle of adjacent joints (A2–BB, B2–CC, C2–DD at 45° and 60°).
3. When the joint dip angle is 75°, a distinct connection from joint ends to the middle of adjacent joints (D1–CC, C1–BB, and B1–AA at 75°) and a more pronounced “tail-to-tail” connection (A2–B2 and B2–C2 at 75°) composite final failure mode are observed.
4. When the joint dip angle is 90°, the failure connection pattern includes both “head-to-head” and “tail-to-tail” connections.

Overall, the variation in the joint dip angle leads to a trend where the final failure mode of the rock bridge evolves from an “end-to-end” connection to a combination of a “head-to-head” connection and “tail-to-tail” connection; the crack initiation points develop from the ends of the original joints toward the middle of the joints and then back toward the joint ends. The final failure mode transitions from being predominantly tensile failure to a combination of tensile and shear failure (for specifics, see the density of shear cracks in the final failure path change in Fig. 11). With the normal stress remaining constant, different inclination angles result in varying lengths of joint connection in the rock bridges, and the longer a rock bridge is, the greater the shear stress required for final failure.

**Figure 12:**
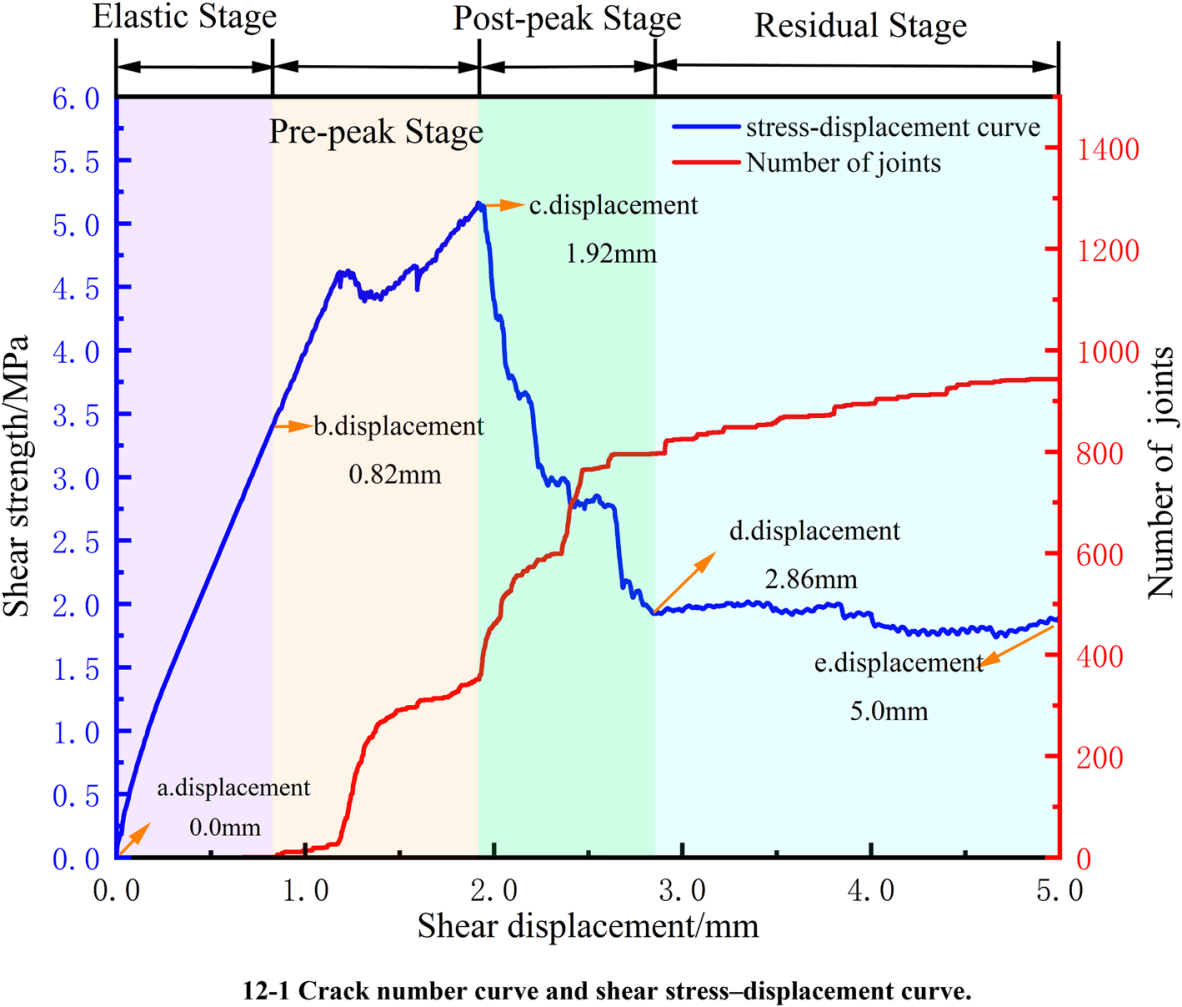

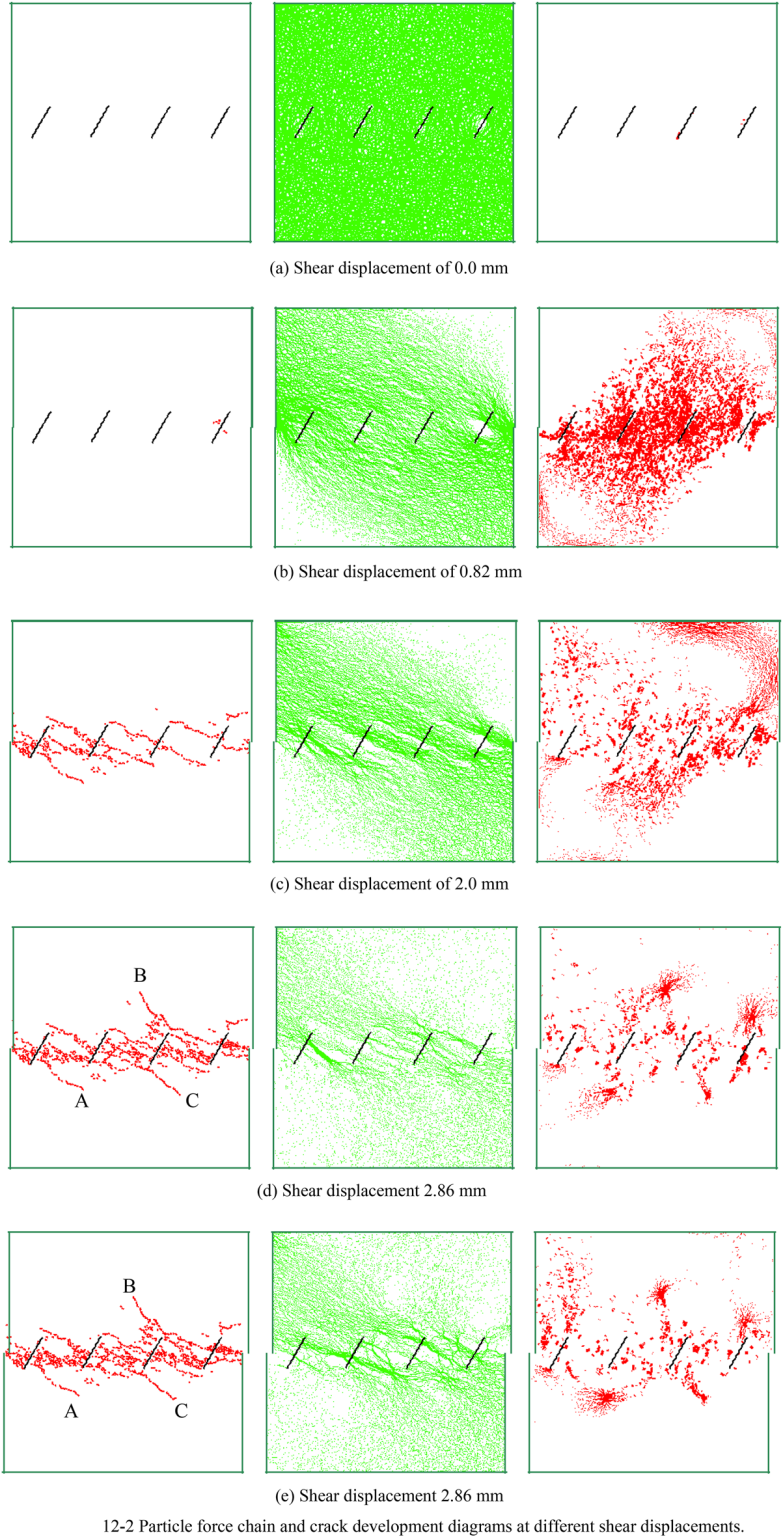
Test results for the 60° joint dip angle.

Figure 12 presents the shear stress‒displacement curve and crack number curve for the specimen with a joint dip angle of 60° (see Fig. 12–1), as well as the crack development and particle force chain diagrams extracted during the direct shear test (see Fig. 12–2, where the first diagram is the crack distribution, the second is the compressive force chain diagram, and the third is the tensile force chain diagram). The shear displacements are 0.0 mm (initial loading stage), 0.82 mm (the starting point of the prepeak stage), 1.93 mm (the point of peak shear stress), 2.86 mm (the starting point of the residual deformation stage), and 5.0 mm (the end point of direct shear).

Combining Figs. 12–1 and 12–2, at the initial loading stage (see Fig. 12-2a), the top and bottom of the specimen are subjected to a pressure of 1 MPa, and the particles are filled with compressive force chains. At this point, the compaction stage of the particles occurs, and the distribution of the compressive force chains is uniform. At the beginning of the prepeak stage (Fig. 12-2b), the shear stress‒displacement curve transitions from linear to nonlinear, and the number of cracks begins to increase. The compressive force chains start to concentrate at the loading end and the fixed end, connecting through the intermediate rock bridge to form a rectangular compressive force chain (from the upper left to the lower right). At this time, the tensile force chains are perpendicular to the compressive force chains (from the lower left to the upper right). The number of developing cracks is minimal, and most are distributed around the original joints. At the peak shear stress point (Fig. 12-2c), the shear stress‒displacement curve decreases rapidly, while the number of developing cracks increases sharply from 300 to 800. A small macroscopic fracture zone is formed at the upper part of the shear plane, and the compressive force chains in the vicinity gradually disappear. The granular force chain diagram shrinks toward the shear surface, the pressure chain at the nodal tip near the loaded and fixed ends becomes larger, and its direction is parallel to the direction of the prefabricated nodal normal. A comparison of the granular force chain diagram with the fracture development diagram shows that under shear pressure, cracks are first produced at the right nodal tip, and as the shear displacement continues to increase, the cracks gradually extend along the edges of the pressure chain to the neighboring nodal sections. It should be noted that the cracks at the nodal tips are formed due to damage under tension. Entering the residual stage (see Fig. 12-2d), the shear stress‒displacement curve and the crack number curve gradually tend to stabilize. The particle force chain diagram indicates that the compressive force chains become thinner and pass through the rock bridge, with the overall tensile force chains gradually disappearing. The concentration of local tensile force chains at three points is the cause of the rock mass developing three cracks (see Fig. 12-2d and (e) A, B, C) that extend deep into the rock mass. At the end of direct shear (see Fig. 12-2e), the shear stress‒displacement curve and the crack number curve show minimal changes. The crack distribution diagram and the tensile force chain diagram remain largely unchanged. Since the specimen is still being loaded during the residual deformation stage, the position of the blocks along the shear band changes, and the compressive force chains concentrated on these blocks exhibit variations during this phase.

The crack development diagram indicates that the damage to the model specimen includes both tensile cracks and shear cracks. Therefore, the damage to the rock body with non-coplanar discontinuous joints develops from the initial tensile damage due to tensile stress concentrating at the tip of the joints to the final tensile‒shear composite damage.

### Effect of the shear displacement rate

**Figure 13:**
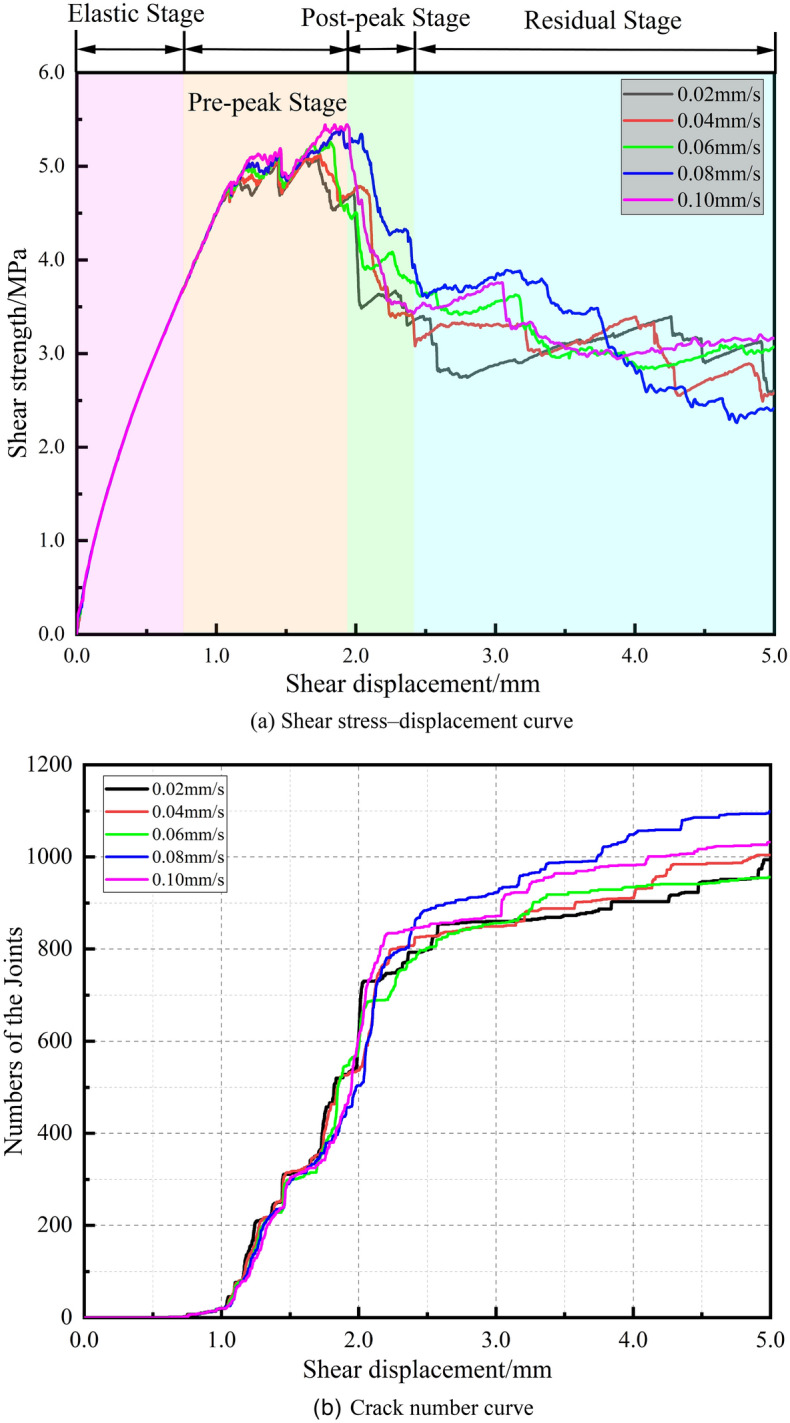
Shear stress‒displacement curves and crack number curves under a range of shear rates.

In this subsection, the normal stress for the five experimental groups is set to 2 MPa, with a joint dip angle of 60°. Figure 13 presents the shear stress‒displacement curves and crack number curves for shear rates ranging from 0.02 to 0.10 mm/s.

Combining Fig. 13a, b, it can be observed that in the linear elastic stage, variations in the shear rate have almost no effect on the shear stress‒displacement curve or the crack number curve, with all five curves overlapping, indicating that no new cracks are generated within the specimen. In the prepeak stage, the trends of the curves change in accordance with the variation in shear rate, with all curves exhibiting a double-peak pattern with the peak shear stress occurring at the second peak. Correspondingly, the crack number curve shows a rapid increase before each peak. In the postpeak stage, all five curves rapidly decrease to the residual stage, with minimal differences in the shear displacement experienced during the postpeak phase, and the total number of cracks generated during this stage also does not vary significantly. In the residual stage, the shear stress generally remains stable, and the cracks generated due to frictional wear vary under different shear rates.

**Figure 14:**
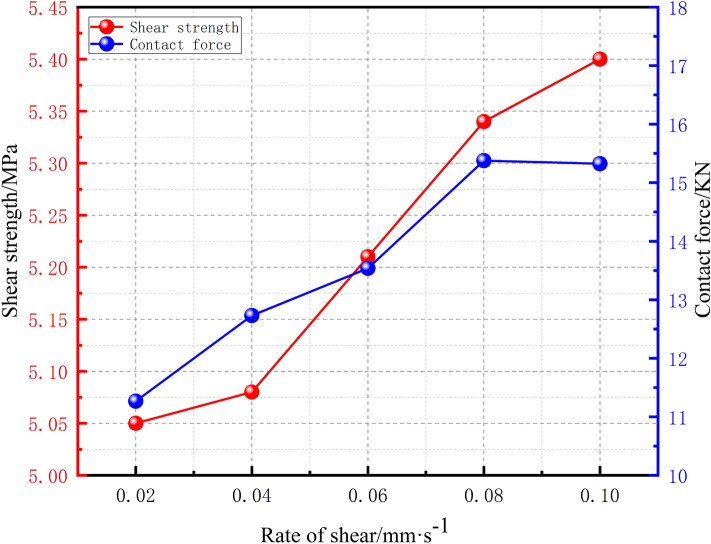
Relationship between the peak shear stress and the particle contact force and shear rate.

Based on Fig. 13, we further deduce the relationship between the peak shear strength of the model specimen and the different shear rates. In Fig. 14, a curve that shows the variation in the particle contact force with the shear rate results is presented. The figure shows that the peak shear strength increases nonlinearly with increasing shear rate. When the shear rate increases from 0.02 to 0.04 mm/s, the shear strength increases by 0.67%, which is the smallest increase at this stage; when the shear rate increases from 0.02 to 0.1 mm/s, the shear strength increases by 6.95%. Overall, the increase in the shear rate is relatively large.

To investigate the intrinsic mechanism of the effect of the shear rate on the peak shear stress, the maximum contact pressure between particles was monitored by the built-in Contact Force Mag module of PFC. As supported by Fig. 14, the particle contact pressure increases with increasing shear rate, and a greater contact pressure increases the degree of interparticle interlocking, which requires a greater shear force for shear damage.

The difference in the results when the shear rate is v = 0.02 versus 0.1 mm/s is most obvious in the elastic phase, and the shear stress curve shows that a high shear rate causes the elastic phase curve to undulate. Entering the residual friction stage, the high-shear-stress curve has no stepwise stress drop but undulates greatly.

## Conclusion

This paper presents multiple numerical simulation experiments on non-coplanar discontinuously jointed rock masses. Macroscopically, it investigates the variations in peak shear stress, crack development, and rock bridge final failure modes under different normal stresses, joint dip angles, and shear rates. Microscopically, the examination of the crack development and force chain diagrams at various stages reveals the mechanisms of rock bridge crack propagation and final failure under direct shear. The main conclusions are as follows:

1. Under the conditions of five levels of normal stress, the peak shear stress, peak displacement, and crack number (especially the total number of cracks generated in the postpeak stage) of the non-coplanar discontinuously jointed rock masses increase nonlinearly with increasing normal stress. A significant climbing phenomenon is observed at 1 MPa and 2 MPa, while the climbing phenomenon and the development of long tensile cracks are suppressed at 3–5 MPa. This results in the concentration of crack development along the shear band connecting the loading end to the fixed end. Additionally, as the normal stress increases, the spatial distribution of the tensile force chains changes, and the initiation sites of new cracks (tensile cracks) shift from the ends of the joints toward the middle of the joints, thereby affecting the final failure mode of the rock bridges within the jointed rock mass. Tensile cracks are suppressed and develop within the shear band area as the normal stress increases, making it difficult for tensile cracks to develop outside the shear band. Consequently, the proportion of tensile cracks decreases, while the proportion of shear cracks correspondingly increases.
2. Under different joint dip angles and normal stresses, during the direct shear process, the variation curves of the peak shear stress with increasing dip angle all exhibit an “S”-shaped nonlinear pattern. Its minimum value occurs at a joint dip angle of 15°, while the maximum value occurs at 65°. Moreover, when the normal stress is between 2 and 5.5 MPa, the curve exhibits a concave downward phenomenon within the 45°–65° dip angle range, and at a normal stress of 5.0 MPa, the peak shear stress occurs in the special case of a joint dip angle of 45°.
3. The joint dip angle has a significant impact on the final failure mode of rock bridges in the rock mass. Specifically, as the joint dip angle increases, the final failure mode of the rock bridges transitions from an “end-to-end” connection to a combination of a “head-to-head” connection and “tail-to-tail” connection.
4. Under different shear rates, the trends of the shear stress‒displacement curves and the crack number curves are the same. The peak shear stress increases from 5.05 to 5.40 MPa as the shear rate varies from 0.02 to 0.10 mm/s, which is an increase of 6.95%. Overall, the shear rate has a certain impact on the peak shear stress, but the impact is not significant.
5. The shear stress‒displacement curve exhibits distinct stages. The spatial distribution of the tensile force chains changes as shearing progresses, and stress concentration occurs at the tips of the original joints, which is the reason for the development of long tensile cracks in the deeper parts.

## Data Availability

The datasets used and/or analyzed during the current study are available from the corresponding author upon reasonable request. All the data generated or analyzed during this study are included in this published article [and its supplementary information files].
